# AlloDerm Sling for Correction of Synmastia After Immediate, Tissue Expander, Breast Reconstruction in Thin Women

**Published:** 2009-11-12

**Authors:** G. Grabov-Nardini, J. Haik, E. Regev, E. Winkler

**Affiliations:** Department of Plastic & Reconstructive Surgery, Sheba Medical Center, Tel-Hashomer, Ramat Gan, Israel (Affiliated to Sackler School of Medicine, Tel-Aviv University, Tel-Aviv, Israel)

## Abstract

**Introduction:** Synmastia is a condition in which the breasts are conjoint and the natural intermammary sulcus is obliterated. It is the rarest type of breast implant malpositioning during breast augmentation; however, it is the most difficult one to correct. AlloDerm is an acellular dermal matrix that is assuming a major role in immediate breast reconstruction in recent years. **Methods:** In the past 2 years, we have treated 3 thin women, a total of 6 breasts, for correction of synmastia after bilateral immediate breast reconstruction, using tissue expanders and skin sparing mastectomy. All of them suffered from synmastia, which manifested immediately after the mastectomy and accelerated during tissue expander inflation. We exchanged the expander into silicone implants, and during the same procedure we corrected the synmastia, using an AlloDerm sling. A thick sheet of AlloDerm (Life-Cell Corp, Branchbung, NJ) is used and the AlloDerm sheet is designed into a long narrow sling. Then, the sling is sutured into place. **Results:** This technique successfully resolved the synmastia. **Conclusion:** The use of an AlloDerm sling to reinforce the capsule and the AlloDerm incorporation into it ensures a sound solution with a low recurrence rate.

Synmastia is a condition in which the breasts are conjoint and the natural intermammary sulcus is obliterated. It is the rarest type of breast implant malpositioning during breast augmentation; however, it is the most difficult one to correct. This condition is caused by migration of the breast implants across the chest midline, medially. The etiology of this rare condition is usually a consequence of overzealous dissection of the implant pocket or the use of excessively oversized implants (400 cc and more) during breast augmentation. Other factors that contribute to the creation of synmastia are subpectoral implant positioning, thoracic deformities, mastopexy augmentation,^1^ congenital synmastia,^2^ and trauma.

Although it is a rare complication after breast augmentation, the rise in immediate breast reconstruction following mastectomy^3^ contributes to the increased incidence of synmastia. During mastectomy, the general surgeon's goal is primarily achieving a tumor-free state, thereby dictating a more aggressive approach, which at times may result in violation of the chest midline resulting in synmastia. Moreover, when tissue expanders are used, inflation can result in thinning of surrounding tissue and in rare cases release of the medial origin of the pectoral muscle and synmastia.

The correction of synmastia is the re-creation of the intermammary sulcus. To restore the sulcus, various surgical techniques are available, including capsuloraphy,^4^ change of implant plane, medial suturing of the deep dermis down to the presternal periosteum, or in extreme cases explanation and delayed secondary augmentation with smaller breast implants. Newer techniques include adjustable implants^5^ or creation of a neosubpectoral pocket.^6^ Regardless of the surgical approach applied, recurrence rates are high.

AlloDerm is an acellular dermal matrix, which is assuming a major role in immediate breast reconstruction in recent years.^7^ The AlloDerm sling is durable, nonimmunogenic, and elastic. It is mainly used not only to cover the lower pole of reconstructed breast implants, but also as an alternative material for static facial slings^8^ or large abdominal defect closure.^9^ An additional advantage is the ability of the AlloDerm sheet to incorporate into the recipient surrounding tissue.^10^ AlloDerm reduces the risk of implant exposure and allows a natural and safe breast reconstruction.

In the past 2 years we have treated 3 thin women, a total of 6 breasts, for correction of synmastia after bilateral immediate breast reconstruction, using tissue expanders and skin sparing mastectomy. All of them suffered from synmastia, which manifested immediately after the mastectomy and accelerated during tissue expander inflation. We used an AlloDerm medial sling to correct the synmastia in the second stage of breast reconstruction, during exchange of the tissue expanders to permanent silicone implants.

## SURGICAL TECHNIQUE

**Figure 1:**
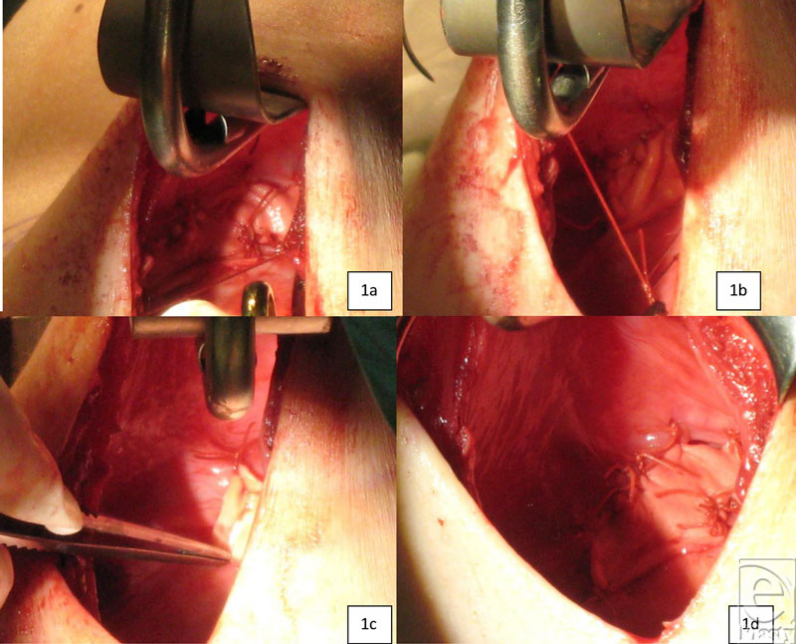
Patient S.A. during the medial AlloDerm sling correction of synmastia. Parts a-c show positioning of the AlloDerm sling and suturing, Rt breast. Parts b-d show that the AlloDerm sling is in place sutured to the 2 leafs of capsule thereby reinforcing the capsule, Rt breast.

The use of an AlloDerm sling for correction of synmastia provides a long-lasting and reliable solution. We must keep in mind that unlike synmastia correction following breast augmentation, in the reconstructive breast, access is preformed through the old scar with a good visibility of the pocket. The patient does not undergo another operation, and the correction of synmastia is carried out with the expander exchange to a permanent silicone implant. Medial capsulotomies are performed 1 cm from the sternal border (Fig 1). A thick sheet of AlloDerm (Life-Cell Corp, Branchbung, NJ) is used and hydrated in saline. The AlloDerm sheet is designed into a long sling of about 2 × 8 cm^2^ following measurements of the area of medial weakness. The dermal side of the AlloDerm is placed toward the 2 leaves of the capsule, on the medial border of the pocket. Then the AlloDerm sling is sutured in place with poliglactin (vicryl) 2/0 around the aligned capsule. The procedure is repeated on the opposite breast. A pressure dressing between the breasts is the final and crucial step for a good and lasting result.

## CASE PRESENTATION

S.A. was a 36-year-old healthy woman. The patient was thin with small breasts, cap A. The patient suffered from invasive ductal carcinoma with negative sentinel node, which was treated with neoadjuvant chemotherapy. She was found to be a carrier of the *BRCA1* gene. The patient underwent immediate breast reconstruction following bilateral mastectomy. Because of the fact that her breasts were small and the patient desire of larger breasts, tissue expanders were inserted. During the operation, an AlloDerm sheet of 16 × 4 cm^2^ to the lower pole of the pocket was used. After the first operation, the tissue expanders (450 cc) were inflated to a total volume of 500 cc with development of synmastia during the inflation. Six months later the patient underwent exchange of the expanders with silicone implants (440 cc) and correction of synmastia with an AlloDerm sling as mentioned above. During her postoperative period the patient was dressed for 2 weeks with a pressure dressing directly over the sternal area to prevent development of a seroma or edema. After follow-up of almost 1 year, there was no recurrence of synmastia.

## SUMMARY

In the past 2 years we treated 3 women with synmastia following immediate breast reconstruction. All of them were thin with small breasts and underwent mastectomy and immediate breast reconstruction using tissue expanders. Although synmastia will remain a very rare phenomenon, the increase in the rate of immediate breast reconstruction and the quest for oncological control will probably increase its incidence in the following years. The risk of synmastia is even greater in thin women with small breasts probably because of the common use of tissue expanders in the breast reconstruction creates a pressure on the capsule in a way that even a small violation of the midline during mastectomy can manifest at the end of the expansion procedure as synmastia. The correction of synmastia is a challenge for the reconstructive plastic surgeon. The use of an AlloDerm sling to reinforce the capsule and the AlloDerm incorporation into it ensure a sound solution with a low recurrence rate.
